# “I'd Rather Be Alone.” Examining the Interactive Effects of Social Proximity and Social Preference on Suicidal Thinking

**DOI:** 10.1111/sltb.70006

**Published:** 2025-02-15

**Authors:** Sarah L. Brown, Lori N. Scott

**Affiliations:** ^1^ Department of Psychology Florida State University Tallahassee Florida USA; ^2^ Department of Psychiatry University of Pittsburgh School of Medicine Pittsburgh Pennsylvania USA

**Keywords:** dynamic structural equation modeling, ecological momentary assessment, social stress, suicide ideation, young adults

## Abstract

**Introduction:**

Emerging and young adulthood is associated with heightened risk for suicide, with interpersonal factors potentially exerting disproportionate effects during this critical life stage. Research examining the interplay of subjective and objective interpersonal factors for suicide ideation (SI) in daily life is limited.

**Methods:**

Dynamic structural equation models were used to analyze ecological momentary assessment data (21 days; 7 semi‐random daily surveys) in a sample of at‐risk young adults (*N* = 140) to test within‐person main and interactive effects of objective social proximity (alone vs. not alone) and subjective social preference (desire to be alone or with others) on SI severity concurrently and prospectively over 2‐h intervals in daily life.

**Results:**

Preferring to be alone (while alone or with others) was associated with intraindividual near‐term increases in SI severity, whereas preferring to be with others (while alone or with others) was associated with near‐term decreases in SI severity.

**Conclusions:**

Being with others can be either a risk or protective factor for near‐term SI severity depending on whether the present company is desired. Considering multiple interpersonal factors combined may be necessary to understand and treat SI as these factors may either buffer or confer greater near‐term risk depending on other factors.

## 1 Introduction

Emerging and young adulthood is a developmental period linked to increased risk for psychopathology (e.g., Gustavson et al. 2018; Substance Abuse and Mental Health Services Administration [SAMSHA] 2023), suicide ideation (SI), and suicidal behaviors (SB; Centers for Disease Control [CDC] 2024). Even prior to the rapid increase in suicide rates in 2021, suicide was the third‐leading cause of death among adults ages 15–24 and the second‐leading cause of death among adults ages 15–34 in 2020 (CDC 2024). Young adults of ages 18–25 had the highest rates of serious suicidal thoughts (13.6%), suicide plans (1.9%), and suicide attempts (2.1%) in the past year compared to other adult age groups (SAMSHA 2023). Emerging and young adulthood involves a focus on self‐identification, instability in work and romantic relationships, and the navigation of complex social relationships (Arnett et al. 2014; Shulman and Connolly 2013; Wood et al. 2018). Consequently, difficulties in social functioning are likely to emerge, solidify, and unduly impact mental health during this developmental window with lasting effects across the lifespan. There is an urgent need for research to identify modifiable interpersonal factors that may disproportionately affect individuals during this critical life stage.

Numerous theories of suicide (Durkheim 1897; Klonsky and May 2015; Van Orden et al. 2010) and substantial empirical evidence implicate the role of interpersonal factors in both risk (e.g., social isolation, negative interpersonal interactions) and resiliency (e.g., social support, feelings of connectedness) for SI and SB (e.g., Hirsch and Barton 2011; Howarth et al. 2020; Moller et al. 2021; Motillon‐Toudic et al. 2022). Extensive evidence indicates that subjective indicators of interpersonal factors such as feelings of loneliness, burden, social rejection, and positive or negative perceptions of one's social interactions are concurrently and prospectively associated with SI and SB (e.g., Czyz et al. 2019; Hallensleben et al. 2019; Jacobucci et al. 2023; Kaurin et al. 2022; Victor et al. 2019). Fewer studies have examined objective indicators of interpersonal factors, but similar patterns have emerged such that indicators of low social connectedness are associated with increased suicide risk (e.g., Calati et al. 2019; Milner et al. 2015; Molaie 2022; Tsai et al. 2015). Social isolation, defined as a “state in which interpersonal contacts and relationships are quantitatively disrupted or non‐existent” (Calati et al. 2019, 661; de Jong Gierveld and Havens 2004), is a well‐studied objective risk factor. Objective indicators of social isolation, including marital status, living alone, low frequency of social relationships, and unemployment, are associated with increased risk for SI and SB (Calati et al. 2019). Other objective interpersonal factors, including number of social ties (Milner et al. 2015) and aspects of social networks (e.g., number of types of social contacts; Molaie 2022), have been implicated in risk for SI and SB. In general, these findings suggest that both objective and subjective indicators of interpersonal factors provide important information about suicide risk; however, results vary across methodological approaches and populations (Chang et al. 2017; Fässberg et al. 2012; Masi et al. 2011; McClelland et al. 2020; Mueller et al. 2022), with few studies integrating both objective and subjective indicators.

In fact, there is research to suggest that objective and subjective indicators of interpersonal factors may provide distinct information and potentially interact to differentially confer risk for SI and SB (e.g., Masi et al. 2011; Mueller et al. 2022). Research on loneliness not only emphasizes the importance of subjective feelings of social connection in relation to psychopathology and suicide risk (Cacioppo et al. 2015; Hawkley and Cacioppo 2010) but also suggests that loneliness reflects a discrepancy between one's preferred and actual social states (Perlman and Peplau 1982). For instance, individuals who live alone and would be objectively considered socially isolated may or may not report subjective feelings of loneliness (Cacioppo et al. 2015; de Jong Gierveld and Havens 2004). Meanwhile, individuals who are not socially isolated may still report feelings of loneliness. Moreover, these objective and subjective interpersonal states are likely to change over the course of hours and days and may account for the dynamic fluctuations observed in SI (e.g., Kleiman et al. 2017; Victor et al. 2019). Research examining short‐term changes in both objective and subjective interpersonal factors as they relate to SI in daily life may be critical to understand the nuanced associations between interpersonal factors and suicidal thinking.

Research examining the social context of suicidal thinking using both objective and subjective indicators of interpersonal factors in daily life is limited. A recent ecological momentary assessment (EMA) study by Parrish et al. (2021) examining suicide risk among individuals with a psychotic disorder found that being alone was concurrently, but not prospectively (at the next time point), associated with greater feelings of thwarted belonging and perceived burden, both of which are theoretical risk factors for SI (Van Orden et al. 2010). In another recent EMA study of psychiatric inpatients, Hallensleben et al. (2020) found that being with others was associated with lower concurrent feelings of thwarted belonging, whereas a greater discrepancy between one's current and desired social proximity state was associated with greater feelings of thwarted belonging. The descriptive results from this study suggest that feelings of thwarted belonging in different social proximity states (alone versus not alone) may vary depending on the desirability of the company at the time; however, the interaction between social proximity and social preference (including whether being alone is associated with greater thwarted belonging when company is desired) was not formally tested. These findings suggest that one's objective social proximity state *and* the desirability or preference for social proximity are associated with theoretical risk factors for SI; however, it remains unclear how each of these factors may directly impact or interact to influence SI in daily life.

The current study utilizes EMA data from a longitudinal study of at‐risk young adults to examine how objective social proximity (i.e., being alone or being with others), subjective social preference (i.e., desire to be alone or with others), and their interaction are dynamically associated with SI severity both concurrently and prospectively over short time periods (i.e., 2 hours). This study aims to clarify the complex roles of interpersonal factors in the dynamic fluctuation of near‐term suicidal thinking, which may inform standard and just‐in‐time intervention approaches. We hypothesized that there would be a main effect of social proximity, such that being with others would generally be protective and associated with concurrent and near‐term decreases in SI severity, whereas being alone would be associated with concurrent and near‐term increases in SI severity. Further, we hypothesized that there would be an interaction between social proximity and social preference such that an incongruence between actual and desired social states (i.e., being with others when preferring to be alone; being alone when preferring to be with others) would represent high‐stress social states associated with concurrent and near‐term increases in SI severity.

## 2 Methods

### 2.1 Participants

The sample consists of 140 young adult men and women between the ages of 18 and 35 (*M*_age_ = 25.34, SD_age_ = 4.64) with SI and/or SB within the past 4 months who were enrolled in a longitudinal study of suicide risk. See Table 1 for detailed demographics and clinical diagnostic information.

**TABLE 1:** Demographic and clinical diagnostic information.

	*n* (%)
Sex assigned at birth	
Male	24 (17.1)
Female	116 (82.9)
Gender	
Cis‐man	21 (15.0)
Cis‐woman	103 (73.6)
Transgender female‐to‐male	2 (1.4)
Transgender male‐to‐female	4 (2.9)
Do not identify as male or female	9 (6.4)
Other	1 (0.7)
Ethnicity	
Non‐Hispanic/LatinX	127 (90.7)
Hispanic/LatinX	13 (9.3)
Race	
Asian	11 (7.9)
Black or African American	22 (15.7)
White	96 (68.6)
More than one race	10 (7.1)
Prefer not to respond	1 (0.7)
Sexual orientation	
Heterosexual/straight	67 (47.9)
Gay/lesbian/homosexual	14 (10.0)
Bisexual	37 (26.4)
Other	16 (11.4)
Not sure	4 (2.9)
Declined	1 (0.7)
Education	
Less than high school or GED	1 (0.7)
High school or GED	11 (7.9)
Some college	47 (33.6)
Technical/trade school certificate	4 (2.9)
College degree or higher	77 (55.0)
Employment	
Not employed	39 (27.9)
Part‐time	46 (32.9)
Full‐time	55 (39.3)
Annual income: M (SD)	$73,546 ($77,751)
Public assistance (e.g., Medicaid, WIC) in last year	49 (35.0)
C‐SSRS SI highest level (past 4 months)	
Wish to be dead	23 (16.4)
Nonspecific active SI	24 (17.1)
Active SI with methods (no plan or intent)	41 (29.3)
Active SI with some intent (no plan)	44 (31.4)
Active SI with specific plan and intent	8 (5.7)
C‐SSRS suicide behavior (past 4 months)	
Actual attempt	7 (5.0)
Interrupted attempt	4 (2.9)
Aborted attempt	7 (5.0)
Preparatory behavior	14 (10.0)
C‐SSRS suicide behavior (lifetime)	
Actual attempt	52 (37.1)
Interrupted attempt	17 (12.1)
Aborted attempt	40 (28.6)
Preparatory behavior	55 (39.3)
SCID‐5‐RV current diagnosis	
Mood disorder	89 (63.6)
Anxiety disorder	91 (65.0)
Obsessive–compulsive disorder	17 (12.1)
Trauma and stress‐related disorder	31 (22.1)
Substance use disorder	39 (27.9)
Eating disorder	8 (5.7)
SCID‐5‐RV lifetime diagnosis	
Mood disorder	139 (99.3)
Anxiety disorder	110 (78.6)
Obsessive–compulsive disorder	29 (20.7)
Trauma and stress‐related disorder	65 (46.4)
Substance use disorder	75 (53.6)
Eating disorder	43 (30.7)
SIDP‐IV current diagnoses	
Borderline personality disorder	20 (14.3)
Antisocial personality disorder	5 (3.6)
Avoidant personality disorder	35 (25.0)

*Note:* *N* = 140. C‐SSRS = columbia suicide severity rating scale (Posner et al. 2011). SCID‐5‐RV = structured clinical interview for DSM‐5 (First et al. 2015). SIDP‐IV = structured clinical interview for DSM‐IV personality disorders (Pfohl et al. 1997), the criteria for which are unchanged in DSM‐5. Percentages may not sum to 100% due to missing data.

### 2.2 Recruitment and Procedures

A total of 150 participants were recruited for a one‐year longitudinal study of suicide risk in an at‐risk transdiagnostic sample of young adult (ages 18–35) men and women. Study recruitment took place from July 2019 through April 2023. Eligibility criteria were as follows: (1) SI at least two times in 1 week or any type of SB in the past 4 months per the Columbia Suicide Severity Rating Scale (C‐SSRS; Posner et al. 2011) and (2) behavioral mental health care at least two times a month at the time of initial recruitment. Participants completed diagnostic interviews followed by a 21‐day web‐based EMA protocol. All participants provided written and oral informed consent, and study procedures were conducted in accordance with the responsible Institutional Review Board.

### 2.3 Measures

#### 2.3.1 EMA Protocol

The 21‐day EMA protocol involved seven semi‐random surveys per day that were scheduled between participants' self‐reported wake and sleep schedules for a maximum of 147 possible prompts. Of the 150 participants enrolled in the larger study, 141 participants started the EMA protocol. One participant was excluded from analyses due to insufficient EMA data, resulting in a final sample of 140. Mean compliance, calculated as percentage of survey prompts received that were completed for each person, was 87%. The median time interval between all assessments was 2.20 hours (*M* = 3.86; Mode = 1.50; SD = 5.50). See Cox et al. (2023) and https://osf.io/f8hge/ for EMA protocol details.

#### 2.3.2 SI Severity

At each EMA prompt, participants rated the following statements intended to capture both passive and active aspects of SI on a scale of 0 (*Not strong at all*) to 100 (*Very strong*): “At this moment, how strong is your… wish to die, wish to live (reverse scored), and wish to die by suicide.” These selected items were derived from the Beck Suicide Scale (Beck and Steer 1991) and have been used in previous EMA studies (e.g., Coppersmith et al. 2019). The average of these three items was calculated at each observation as an index of momentary SI severity. Coefficient omega (*ω*) indicated satisfactory within‐person internal consistency reliability for this scale (*ω* = 0.73). The intraclass correlation coefficient (ICC) for the SI severity scale was 0.69, indicating that 69% of the variability reflects between‐person differences, and the remaining 31% can be attributed to within‐person variance (i.e., differences within the same individual over time) and measurement error.

#### 2.3.3 Social Proximity and Social Preference

At each EMA prompt, participants indicated whether they were currently alone or with others as a measure of momentary social proximity. If they were alone, they were asked to rate how much they would rather be with others on a scale from 1 (*Not at all* ) to 7 (*Extremely* ). If they were with others, they were asked to rate how much they would rather be alone using the same scale. These two items were combined into a momentary social preference score that captures the discrepancy between current and desired states of social proximity with higher scores indicating a stronger preference to be in a different social proximity state. Across observations, participants reported currently being with others 55.4% of the time. When with others, participants reported being with 1–6 other types of individuals (*M* = 1.35; SD = 0.62). Across observations, participants were most frequently with a romantic partner (35.1%), followed by multiple types of others (28.5%), other family member (besides romantic partner or child; 17.5%), co‐worker/classmate (13.9%), friend (11.9%), and others (e.g., roommate, boss/teacher, therapist/doctor, acquaintance, stranger; 13.7%).

### 2.4 Data Analytic Strategy

To test the hypotheses, we used 2‐level dynamic structural equation models (DSEMs) using Bayesian Markov chain Monte Carlo (MCMC) estimation in Mplus version 8.10 (Muthén and Muthén 2023). Momentary data (7 daily prompts; Level 1) were nested within 140 participants (Level 2). For variables that were modeled on both within‐ and between‐person levels, latent mean centering was used to automatically decompose the data into within‐and between‐person effects. The TINTERVAL command was used to account for unequal intervals between observations due to random sampling or missed assessments. This approach rescales time into user‐specified intervals by inserting missing data fields at this chosen interval when there are no observed data. The result is that autoregressive and other time‐lagged regression effects will maintain a constant temporal interpretation even with unequally spaced observations (for additional details, see McNeish and Hamaker 2020). We specified the time interval of interest for time‐lagged associations as 2 hours based on the median time between assessments (2.2 hours), interpretability from a theoretical perspective, and information provided in the Mplus output (which informs the user if the chosen interval does not closely match the actual data). To adjust for linear trends in SI severity across the EMA period, we included time elapsed since the first EMA assessment (“days,” person‐mean centered to aid interpretability of temporal trends after accounting for between‐person differences in number of days of EMA data) as a within‐person covariate. Models were estimated using default noninformative priors, 50,000 iterations, and two MCMC chains. We examined trace plots for irregularities (e.g., trends and cycles) and proportional scale reduction (PSR) values to assess model convergence. Final PSR values for the DSEMs ranged from 1.001 to 1.007, indicating good convergence.

Given our specific interest in within‐person changes in SI severity due to within‐person variability in social proximity and preference (rather than differences in SI severity between individuals based on average social proximity and preference across the EMA period), regression effects were specified only at the within‐person level and were estimated as random effects (i.e., allowed to vary between individuals). We estimated all correlations between random means and slopes on the between‐person level. Prior to inclusion in the moderation models, the interaction term (Social Proximity × Social Preference) was calculated using a 2‐step process to ensure that it accurately captures latent‐mean‐centered social preference at the within‐person level (see Speyer et al. 2023). Social proximity (being alone vs. with others) was not centered as we were interested in examining effects when alone vs. with others as momentary categorical states (rather than adjusting these according to person‐level averages).

To identify potential covariates, we examined bivariate correlations between SI severity (person‐level mean across all observations) and between‐person factors (i.e., age, sex, sexual orientation, gender, ethnicity, race, receipt of public assistance tied to low income, current diagnosis of major depressive disorder per the Structured Clinical Interview for DSM‐5) (First et al. 2015), participation pre‐ vs. post‐COVID‐19, and lifetime suicide attempt history per the C‐SSRS (Posner et al. 2011). None of the correlations were significant (all *p*'*s >* 0.05); however, lifetime suicide attempt history was included as a between‐person covariate given *p* = 0.053. Momentary depressed mood (0 [*Not strong at all*] to 100 [*Very strong*]) was also included as a time‐varying covariate at the within‐ and between‐person level, which allows us to examine the unique contributions of social proximity and social preference to SI severity above and beyond concurrent momentary depressed mood. Our models also adjusted for SI severity at the last time point (*t*‐1), enabling us to draw conclusions about within‐person changes in SI severity from one occasion to another.

Given that social proximity and preference may influence SI severity in the moment and in the near term (2 hours later), separate models were tested to estimate both concurrent and lagged associations. Prior to testing the moderation models specified below, we tested main effects models (contemporaneous and time‐lagged), including social proximity (“with others” coded as 1) and social preference, without the interaction term. For the contemporaneous (concurrent) models, we specified social proximity, social preference, and their interaction (Social Proximity × Social Preference) at *t* predicting within‐person change in SI severity at the same time point (*t*) after adjusting for SI severity at the previous time point (*t*‐1) and concurrent depressed mood (*t*). For the time‐lagged models, we specified social proximity, social preference, and their interaction (Social Proximity × Social Preference) at the last time point (*t*‐1) predicting within‐person change in SI severity at the next time point (*t*) after adjusting for SI severity and depressed mood at the previous time point (*t*‐1). For significant interactions, simple slopes were examined. To best capture the regions of significance of the simple slopes within the possible range of latent‐mean‐centered within‐person social preference scores (−1.56 to 4.44), simple slopes were calculated in increments of 0.25 SD above (+1 SD, +1.25 SD, +1.5 SD) and below (−0.75 SD, −0.5 SD) the mean. We also tested the statistical significance of the difference between the simple slopes (see Paternoster et al. 1998). For the moderation models, separate models were estimated with social proximity coded to reflect both alone and being others as the reference group to understand the effects of social preference relative to social proximity state.

## 3 Results

Descriptive statistics and associations between person‐level study variables are shown in Table 2. Results from the contemporaneous and lagged DSEMs are shown in Tables 3, 4, 5.

**TABLE 2:** Descriptive statistics and bivariate correlations at the between‐person level (*N* = 140).

Variable	1	2	3	4	5	6
1. SI severity	—					
2. Social proximity	−0.23**	—				
3. Social preference	0.22**	−0.03	—			
4. Depressed mood	0.65**	−0.09	0.24**	—		
5. Days	−0.09	0.04	0.11	0.08	—	
6. Suicide attempt status	0.16	−0.09	−0.02	−0.01	−0.11	—
%/M	17.62	—	2.58	33.32	9.78	—
SD	16.37	—	0.76	24.08	2.05	—
Range	0.02–69.94	0–1	1.08–5.91	0.11–98.91	1.32–21.41	0–1
Skewness	0.80	—	0.84	0.65	−0.56	—
Kurtosis	−0.18	—	0.21	−0.30	13.43	—

*Note:* SI severity = momentary suicide ideation severity. Days = time elapsed since the first EMA assessment. Social proximity (0 = alone, 1 = with others). Suicide attempt status (0 = no previous suicide attempt; 1 = one or more previous suicide attempt). Repeated measures from EMA were aggregated by person by calculating the mean across assessments.

** *p* < 0.01.

**TABLE 3:** Main effects of results for the effects of social proximity and social preference on suicide ideation severity.

	Predictor	Standardized estimate	Posterior SD	95% CI (lower, upper)
Contemporaneous model	Criterion: SI severity at (*t*)			
Level 1	SI severity (*t*‐1)	**0.198**	0.010	0.179, 0.218
	Social preference (*t*)	**0.076**	0.008	0.061, 0.091
	Social proximity (*t*)	**−0.041**	0.008	−0.056, −0.025
	Days	**−0.024**	0.007	−0.038, −0.009
	Depressed mood (*t*)	**0.352**	0.010	0.332, 0.370
	Residual variance	**0.684**	0.006	0.672, 0.696
Lagged model	Criterion: SI severity at (*t*)			
Level 1	SI severity (*t*‐1)	**0.265**	0.011	0.243, 0.285
	Social preference (*t*‐1)	0.011	0.009	−0.005, 0.028
	Social proximity (*t*‐1)	−0.016	0.009	−0.033, 0.001
	Days	**−0.031**	0.008	−0.047, −0.016
	Depressed mood (*t*‐1)	**0.110**	0.010	0.091, 0.130
	Residual variance	**0.781**	0.006	0.768, 0.792

*Note:* CI = credibility interval. Days = time elapsed since the first EMA assessment. SI severity = suicide ideation severity. Social proximity (0 = alone, 1 = with others). Estimates are within‐level standardized estimates averaged over clusters. Bolded estimates are significant (i.e., 95% CIs do not cross zero).

### 3.1 Main Effects Models (Table 3)

As hypothesized, we observed a significant main effect of social proximity (Std. est. = −0.041), such that being with others (coded 1) was associated with *decreased* concurrent SI severity. There was also a significant main effect of social preference predicting concurrent SI severity (Std. est. = 0.076), such that preferring to be in a social context that is different from one's current social proximity state was associated with *increased* concurrent SI severity. In the lagged model, there was not a significant main effect of social proximity (Std. est. = −0.016) or social preference (Std. est. = 0.011) on near‐term change in SI severity 2 hours later. Being alone and preferring to be in a different social proximity state were independently associated with increased SI severity in the moment but not 2 hours later.

### 3.2 Moderation Models

#### 3.2.1 Being With Others (Relative to Being Alone; Table 4)

**TABLE 4:** Moderation results for the effects of being with others and preference to be alone on suicide ideation severity.

	Predictor	Standardized estimate	Posterior SD	95% CI (lower, upper)
Contemporaneous model	Criterion: SI severity at (*t*)			
Level 1	SI severity (*t*‐1)	**0.196**	0.010	0.177, 0.215
	Social preference (*t*)	−0.010	0.013	−0.037, 0.014
	Social proximity (*t*)	**−0.036**	0.008	−0.051, −0.020
	Social proximity (*t*) *×* social preference (*t*)	**0.113**	0.013	0.089, 0.139
	Days	**−0.024**	0.007	−0.039, −0.010
	Depressed mood (*t*)	**0.343**	0.009	0.324, 0.361
	Residual variance	**0.675**	0.006	0.662, 0.687
Lagged model	Criterion: SI severity at (*t*)			
Level 1	SI severity (*t*‐1)	**0.258**	0.011	0.237, 0.280
	Social preference (*t*‐1)	**−0.044**	0.018	−0.083, −0.011
	Social proximity (*t*‐1)	−0.012	0.010	−0.031, 0.008
	Social proximity (*t*‐1) *×* social preference (*t*‐1)	**0.062**	0.016	0.030, 0.095
	Days	**−0.033**	0.008	−0.048, −0.018
	Depressed mood (*t*‐1)	**0.108**	0.010	0.088, 0.127
	Residual variance	**0.772**	0.006	0.759, 0.784

*Note:* CI = credibility interval. Days = time elapsed since the first EMA assessment. SI severity = suicide ideation severity. Social proximity (0 = alone, 1 = not alone). Estimates are within‐level standardized estimates averaged over clusters. Bolded estimates are significant (i.e., 95% CIs do not cross zero).

As hypothesized, we observed a significant interaction between social proximity and social preference predicting concurrent SI severity (Std. est. = 0.113) such that being with others (coded 1) was associated with *decreased* concurrent SI severity when preference to be alone was low (at least −0.50 SD below the mean; Unstd. est. = −1.702, SD = 0.281, *p* < 0.05). However, being with others was associated with *increased* concurrent SI severity when preference to be alone was high (at least +1 SD; Unstd. est. = 1.015, SD = 0.321, *p* < 0.05).

Results were similar for the lagged model, showing that the interaction between social proximity and social preference (Std. est. = 0.062) was prospectively associated with near‐term increases in SI severity; however, these differences were only observed at more extreme deviations from the mean of social preference. As hypothesized, being with others was associated with *decreased* near‐term SI severity 2 hours later when preference to be alone was low (at least −0.75 SD; Unstd. est. = −0.962, SD = 0.479, *p* < 0.05). Being with others was associated with *increased* near‐term SI severity 2 hours later when preference to be alone was high (at least +1.25 SD; Unstd. est. = 0.929, SD = 0.526, *p* < 0.05). Together these findings show that being with others was associated with *decreased* SI severity in the moment and 2 hours later when company was desired, whereas being with others but desiring to be alone was associated with *increased* SI severity in the moment and 2 hours later.

#### 3.2.2 Being Alone (Relative to Being With Others; Table 5)

**TABLE 5:** Moderation results for the effects of being alone and preference to be with others on suicide ideation severity.

	Predictor	Standardized estimate	Posterior SD	95% CI (lower, upper)
Contemporaneous model	Criterion: SI severity (*t*)			
Level 1	SI severity (*t*‐1)	**0.196**	0.010	0.178, 0.215
	Social preference (*t*)	**0.145**	0.011	0.122, 0.166
	Social proximity (*t*)	**0.041**	0.008	0.025, 0.056
	Social proximity (*t*) × social preference (*t*)	−**0.098**	0.012	−0.121, −0.075
	Days	**−0.025**	0.008	−0.039, −0.010
	Depressed mood (*t*)	**0.344**	0.009	0.325, 0.362
	Residual variance	**0.677**	0.006	0.664, 0.689
Lagged model	Criterion: SI severity at (*t*)			
Level 1	SI severity (*t*‐1)	**0.259**	0.011	0.238, 0.281
	Social preference (*t*‐1)	**0.046**	0.013	0.020, 0.070
	Social proximity (*t*‐1)	**0.019**	0.009	0.002, 0.036
	Social proximity (*t*‐1) *×* social preference (*t*‐1)	−**0.050**	0.013	−0.076, −0.024
	Days	**−0.031**	0.008	−0.048, −0.017
	Depressed mood (*t*‐1)	**0.107**	0.010	0.089, 0.128
	Residual variance	**0.774**	0.006	0.762, 0.785

*Note:* CI = credibility interval. Days = time elapsed since the first EMA assessment. SI severity = suicide ideation severity. Social proximity (1 = alone, 0 = with others). Estimates are within‐level standardized estimates averaged over clusters. Bolded estimates are significant (i.e., 95% CIs do not cross zero).

As hypothesized, we observed a significant interaction between social proximity and social preference predicting concurrent SI severity (Std. est. = −0.098). However, the direction of the interaction was in the opposite direction from our hypotheses such that being alone (coded 1) was associated with *increased* concurrent SI severity when preference to be with others was low (at least −0.50 SD; Unstd. est. = 1.749, SD = 0.274, *p* < 0.05). However, being alone was associated with *decreased* concurrent SI severity when preference to be with others was high (at least +1 SD; Unstd. est. = −0.828, SD = 0.323, *p* < 0.05).

Results were similar for the lagged model, showing the interaction between social proximity and social preference (Std. est. = −0.050). Contrary to our hypothesis, being alone was associated with *increased* near‐term SI severity 2 hours later when preference to be with others was low (at least −0.50 SD; Unstd. est. = 0.859, SD = 0.389, *p* < 0.05). However, being alone was associated with *decreased* SI severity 2 hours later when preference to be with others was high (at least +1.5 SD; Unstd. est. = −0.904, SD = 0.435, *p* < 0.05). These findings show that being alone was associated with *increased* SI severity in the moment and 2 hours later when company was not desired, whereas being alone but still strongly desiring to be in the company of others was associated with *decreased* SI severity in the moment and 2 hours later.

#### 3.2.3 Simple Slopes Comparisons Across Being With Others and Alone Models

To determine if a strong preference to be alone or with others was differentially associated with near‐term changes in SI severity, depending on social proximity state, we tested the difference between simple slopes across Being with Others and Being Alone models. The simple slopes of being with others and strongly preferring to be alone (+1.5 SD) and being alone and strongly preferring to be alone (−0.5 SD) were not significantly different in either the concurrent (*z* = 0.35, *p* = 0.726, two‐tailed) or the lagged models (*z* = 0.44, *p* = 0.660, two‐tailed). In other words, both social states were similarly associated with near‐term increases in SI severity. The simple slopes of being with others and strongly preferring to be with others (−0.5 SD) and being alone and strongly preferring to be with others (+1.5 SD) were not significantly different in either the concurrent (*z* = −0.03, *p* = 0.974, two‐tailed) or the lagged models (*z* = 0.30, *p* = 0.76, two‐tailed). In other words, both social states were similarly associated with near‐term *decreases* in SI severity.^1^

## 4 Discussion

Emerging and young adulthood is associated with heightened risk for SI and SB (e.g., SAMSHA 2023; CDC 2024), with interpersonal factors potentially exerting disproportionate effects during this critical life stage (e.g., Wood et al. 2018). The research examining both subjective and objective interpersonal factors for SI in daily life is limited, with two prior EMA studies (Hallensleben et al. 2020; Parrish et al. 2021) providing support for the role of objective social proximity and subjective social preference in risk for SI. However, these studies did not directly examine SI or the potential that objective and subjective interpersonal factors may interact to differentially confer risk for SI. To address these critical gaps in the literature, we used a DSEM approach with EMA data from young adults with recent SI or SB to examine the independent and interactive effects of objective social proximity and subjective social preference on momentary and near‐term SI severity over 2‐hour intervals in daily life. Our study provides direct evidence that social proximity, social preference, and their interaction are proximally associated with near‐term changes in SI severity.

First, we examined the independent effects of social proximity (i.e., being alone vs. with others) and social preference (i.e., desire to be with others [if alone] or desire to be alone [if with others]) on SI severity. As hypothesized, being with others was associated with decreases in SI severity in the moment, but this association was not significant in the time‐lagged model (i.e., 2‐hours later). Further, preferring to be in a social proximity state that is inconsistent with one's current state (i.e., desire to be with others [if alone] or desire to be alone [if with others]) was associated with momentary, but not prospective, increases in SI severity. These findings are consistent with previous EMA studies that found a greater discrepancy between current and desired social proximity states and being alone were independently and concurrently, but not prospectively, associated with theoretical risk factors for SI (Hallensleben et al. 2020; Parrish et al. 2021). The current study extends these previous findings in two important ways: (1) examining SI itself rather than theoretical risk factors and (2) examining both concurrent and near‐term (time‐lagged) associations while adjusting for previous SI severity and time‐varying depressed mood, thereby elucidating changes in SI as a function of the predictors of interest.

Second, we examined the interaction between social proximity and social preference on momentary and near‐term SI severity, given prior evidence that these states may interact to impact risk for suicidal thinking (e.g., Masi et al. 2011; Mueller et al. 2022). We hypothesized that an incongruence between actual and desired social states (i.e., being with others when desiring to be alone; being alone when desiring to be with others) would represent high‐stress social states associated with increases in SI severity in the moment and 2 hours later. Our results were partially consistent with our hypotheses, such that being with others when preferring to be alone was concurrently and prospectively associated with *increased* SI severity, whereas being with others when preferring to be with others was concurrently and prospectively associated with *decreased* SI severity. Our findings show that being with others can either be a near‐term risk or protective factor for short‐term changes in SI severity depending on whether the present company is desired. Being with others when desired may mitigate SI severity in the moment and for the next couple of hours. However, being with others and wanting to be alone may exacerbate SI severity in the moment and over the next couple of hours.

Contrary to our hypotheses, we found that being alone and *still* desiring to be with others was associated with *decreased* (rather than increased as hypothesized) SI severity in the moment and 2 hours later. Conversely, being alone and wanting to be alone was associated with *increased* SI severity in the moment and had lasting effects for the next couple of hours. Thus, whether objective social proximity functions as a risk or protective factor for SI depends on individuals' social preferences in that moment. Comparisons across models further support that strongly preferring to be with others regardless of actual social proximity (alone or with others) appears to be protective against worsening SI in the near‐term. This preference may be an indicator for a number of interpersonally based protective factors for SI, including a strong sense of belonging, strong social support networks, a sense of connectedness, or expectations regarding future positive or supportive social interactions. However, strongly preferring to be alone was associated with worsening SI, irrespective of objective social proximity. Together, our findings suggest that merely being in the presence of others is not necessarily always protective against suicidal thinking and being alone may not always be associated with increases in suicidal thinking.

The EMA protocol for this study involved frequent semi‐random surveys (seven times a day) assessing both subjective and objective interpersonal factors in an at‐risk sample of young adults. This allowed us to capture and model dynamic changes in SI and interpersonal factors over short time periods (2 hours), which is a strength given mounting evidence for dynamic fluctuations in SI over short timescales (e.g., Coppersmith et al. 2023; Kleiman et al. 2017). While the current study focused on social proximity and social preference, incorporating additional interpersonal factors may improve our understanding of the social context of suicide risk. For example, the perceived quality of the social interactions themselves (e.g., supportive or negative) may impact momentary social preferences. However, our finding that being alone and desiring to be alone was associated with increases in SI severity may be more consistent with a conceptualization of hopelessness about social interactions rather than the direct impact of the social interactions themselves (since no interaction occurred). We speculate that poorer quality social interactions may distally impact the degree to which social proximity states and social preference vary between individuals, such that individuals who tend to have poorer quality social interactions may reduce engagement in social interactions with others and develop a more stable preference to be alone over time. However, poorer quality social interactions are likely only to exert a weak impact on short‐term within‐person fluctuations in social proximity and social preference except perhaps in the case of significant social stressors, yet this remains untested. Alternatively, a desire to be alone may also reflect or be driven by feelings of burden, a proximal risk factor for SI emphasized by the interpersonal theory of suicide (Van Orden et al. 2010). It is plausible that individuals who believe their presence or life makes things worse for others may prefer to isolate to spare others from the perceived burden of their company. This interpretation is also consistent with the previous finding by Parrish et al. (2021) that being alone was associated with greater feelings of burden. Further, advancements in passive sensing offer additional approaches to assessing social proximity states (e.g., Janssen et al. 2024) that could be integrated with EMA‐based approaches. Future EMA studies specifically designed to capture near‐term fluctuations in other subjective and objective interpersonal factors in demographically and clinically diverse samples may prove useful.

In this study, we chose to capture a broad range of suicidal thinking by quantifying SI severity as a composite variable of wish to die, wish to live (reverse scored) and desire to die by suicide. This approach has been used in previous EMA studies on suicidal thinking (e.g., Coppersmith et al. 2019), and the acceptable within‐person internal consistency of SI severity in this study suggests that these items may reflect a common underlying SI severity factor in our sample. Further, multi‐item assessments tend to be more reliable and less susceptible to the influence of imprecise language, although single‐item assessments of suicide risk may have utility (e.g., Ammerman et al. 2021; Gratch et al. 2022). A potential limitation of this approach is the inability to draw conclusions about specific types of SI given the conceptual differences between active and passive SI (e.g., Silverman et al. 2007; Van Orden et al. 2010). It is also possible that in the case of suicidal intent and SB, social proximity regardless of social preference may be protective against SB, at least in the moment. Future research examining how social proximity and social preference may impact active SI specifically, as well as other suicide‐related outcomes such as intent or types of SB, is worthwhile.

The results of the current study suggest that treatment approaches should consider the combination of subjective and objective interpersonal factors, as treatments targeting only one may be insufficient to reduce suicide risk (e.g., Masi et al. 2011; Mueller et al. 2022). If these findings replicate, our results suggest that merely promoting social contact could have the iatrogenic effect of worsening SI severity depending on if being with others in the moment is desired. Prior to suggesting increased social interaction, it would be important to understand whether a person generally desires to be with others and the degree to which this desirability fluctuates, which may also depend on additional factors that have yet to be examined (e.g., quality of social relationships, type of relationship). Addressing practical barriers (e.g., identifying opportunities to have positive social interactions, decreasing the frequency of negative social interactions) may be beneficial among individuals who generally prefer to be with others. On the other hand, targeting the desirability of social interactions (e.g., cognitive restructuring of hopelessness related to social interactions or feelings of burden or thwarted belonging) may be more effective, as our findings suggest that strongly preferring to be with others is protective regardless of whether someone is alone or with others. Consistent with the view that the need to belong is fundamental for well‐being (Baumeister and Leary 1995), cultivating a stable sense of belonging that can withstand fluctuations in social proximity—often influenced by factors such as the availability of others and location—may help protect against short‐term increases in SI. Prior to drawing firm conclusions regarding implications for standard treatments or the development of just‐in‐time interventions, more research examining the interplay of subjective and objective interpersonal factors on risk and resiliency for SI and SB in daily life across various samples is needed.

## 5 Conclusions

Findings from the current study solidify the need to examine the interplay of subjective and objective interpersonal factors as they relate to dynamic fluctuations in suicide risk in daily life across diverse samples, which would inform standard and just‐in‐time treatment approaches.

## Ethics Statement

The University of Pittsburgh approved this study on October 28, 2019, and was assigned #STUDY18100158. All participants provided voluntary consent before participating in research activities.

## Conflicts of Interest

The authors declare no conflicts of interest.

## Data Availability

Detailed results and analysis code are publicly available on the Open Science Framework at https://osf.io/f8hge/. Deidentified data used to produce these results are available directly from the corresponding author upon request.

## Endnotes

^1^
We also estimated models for each individual SI indicator (wish to die, wish to live [reverse scored], desire to die by suicide) as the dependent variable independently and results showed a consistent pattern of findings that did not differ substantially and generally converged on the same interpretations as those reported based on the composite SI severity score.
